# Complementation between Two Tospoviruses Facilitates the Systemic Movement of a Plant Virus Silencing Suppressor in an Otherwise Restrictive Host

**DOI:** 10.1371/journal.pone.0044803

**Published:** 2012-10-16

**Authors:** Sudeep Bag, Neena Mitter, Sahar Eid, Hanu R. Pappu

**Affiliations:** 1 Department of Plant Pathology, Washington State University, Pullman, Washington, United States of America; 2 Queensland Alliance for Agriculture and Food Innovation, University of Queensland, St. Lucia, Queensland, Australia; National Institute of Health, United States of America

## Abstract

**Background:**

New viruses pathogenic to plants continue to emerge due to mutation, recombination, or reassortment among genomic segments among individual viruses. Tospoviruses cause significant economic damage to a wide range of crops in many parts of the world. The genetic or molecular basis of the continued emergence of new tospoviruses and new hosts is not well understood though it is generally accepted that reassortment and/or genetic complementation among the three genomic segments of individual viruses could be contributing to this variability since plants infected with more than one tospovirus are not uncommon in nature.

**Methodology/Principal Findings:**

Two distinct and economically important tospoviruses, *Iris yellow spot virus* (IYSV) and *Tomato spotted wilt virus* (TSWV), were investigated for inter-virus interactions at the molecular level in dually-infected plants. Datura (*Datura stramonium*) is a permissive host for TSWV, while it restricts the movement of IYSV to inoculated leaves. In plants infected with both viruses, however, TSWV facilitated the selective movement of the viral gene silencing suppressor (NSs) gene of IYSV to the younger, uninoculated leaves. The small RNA expression profiles of IYSV and TSWV in single- and dually-infected datura plants showed that systemic leaves of dually-infected plants had reduced levels of TSWV N gene-specific small interfering RNAs (siRNAs). No TSWV NSs-specific siRNAs were detected either in the inoculated or systemic leaves of dually-infected datura plants indicating a more efficient suppression of host silencing machinery in the presence of NSs from both viruses as compared to the presence of only TSWV NSs.

**Conclusion/Significance:**

Our study identifies a new role for the viral gene silencing suppressor in potentially modulating the biology and host range of viruses and underscores the importance of virally-coded suppressors of gene silencing in virus infection of plants. This is the first experimental evidence of functional complementation between two distinct tospoviruses in the *Bunyaviridae* family.

## Introduction

The virus family *Bunyaviridae* consists of five genera and viruses with negative-stranded RNA genomes. All but one genera cause serious diseases in animals and humans [1]. Only one genus, *Tospovirus*, in this family consists of viruses that infect plants. Tospoviruses cause some of the most destructive diseases in important crop plants and world-wide annual losses could be as high as U.S. $1 billion [2]. Tospoviruses are delineated to the species level based on serological relationships and N gene sequence homologies among the viruses. *Tomato spotted wilt virus* (TSWV) was the first tospovirus described and is one of the most-studied tospoviruses [3]–[5]. Tospoviruses are transmitted by thrips (Thysanoptera: Thripidae) in a persistent and propagative manner [6]–[7], and only about 10 of the 5500 known species of Thysanoptera are reported to be vectors of tospoviruses [8].

For many years, genus *Tospovirus* was thought to be a monotypic genus consisting of only TSWV until a second tospovirus, *Impatiens necrotic spot virus* (INSV), was described [9]. Currently more than 20 distinct tospoviruses are reported from different parts of the world [2], [10]). In the US, besides TSWV and INSV, *Iris yellow spot virus* (IYSV) was the third tospovirus to be reported [11]. Most recently, two more tospoviruses, *Groundnut ring spot virus* (GRSV) in tomato in Florida [12] and Soybean vein necrosis-associated virus (SVNaV) in soybean in the Midwestern and midsouthern states of the USA [13] were described in the USA.

The increasing diversity of tospoviruses has been attributed to shifts in crop production, differing specificity of thrips vectors, and the segmented nature of the viral genome which could result in genetic reassortants. Moreover, it is generally accepted that tospoviruses, like other RNA viruses, show high mutation rates because of the lack of proofreading ability of their replicases [14]. Genetic reassortment among genomic components of viruses with divided genomes is known to take place in nature [15]. This is likely to contribute to the natural variation and subsequent evolution of viral genomes as in the case of segmented, positive-stranded RNA viruses, bromoviruses, cucumoviruses and nepoviruses [16]. Viruses with segmented RNA genomes use reassortment as a mechanism of genetic divergence and virus variability, *i.e.* the exchange of genomic segments among different parents. This is facilitated by the presence of mixed infections in plants under natural conditions.

The genome of tospoviruses consists of three RNAs, large (L), medium (M) and small (S). The L RNA is organized in negative sense orientation, whereas the M and S RNAs are in ambisense [17], [5]. The L RNA codes for the RNA dependent RNA polymerase (RdRp) in negative sense, the M RNA codes for a nonstructural protein, NSm, in sense direction and the glycoprotein precursor (G_N_/G_C_) in antisense orientation. The S RNA codes for a non-structural protein (NSs) in sense direction and the nucleocapsid protein (N) in antisense direction. The NSm and G_N_/G_C_ proteins coded by the M RNA play important roles in cell to cell virus movement in plant host, and in vector transmission, respectively [18], [19]. The N protein and NSs protein encoded by the S RNA serve as the nucleoprotein and suppressor host-mediated gene silencing, respectively [3]–[5], [20].

The segmented genome of tospoviruses facilitated genetic reassortment studies to map genetic determinants for symptomatology [21], virus resistance [22], and thrips transmission [19]. These studies utilized isolates of the same virus and the reassortments were the result of intra-species exchange of genomic RNAs.

**Figure 1:**
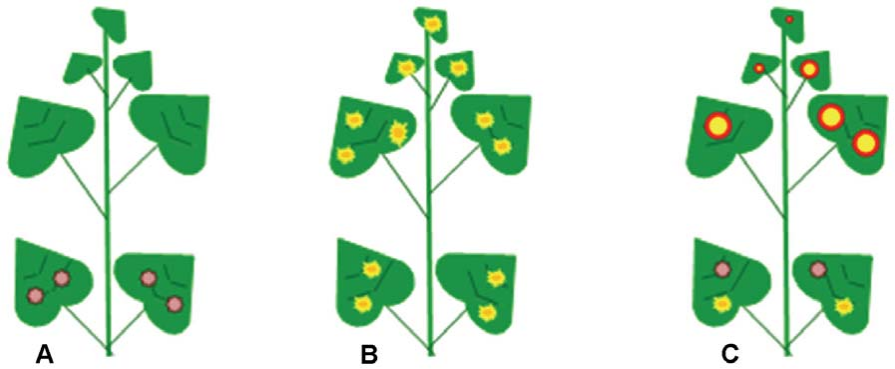
Schematic representation of *Datura stramonium* as a differential host to *Iris yellow spot virus* (IYSV) and *Tomato spotted wilt virus* (TSWV). (A) IYSV infection is localized in inoculated leaves; (B) TSWV causes systemic infection; (C) Co-inoculation of IYSV and TSWV results in severe systemic infection.

Mixed infections of two distinct tospoviruses in the same host plant have been known to occur in commercial production systems. Kunkalikar et al. [23] reported the mixed infection of watermelon (*Citrullus lanatus*) plants by *Groundnut bud necrosis virus* (GBNV) and *Watermelon bud necrosis virus* (WBNV) under field conditions in central India. Similarly, TSWV and IYSV were shown to infect and co-exist in onion plants under field conditions in Georgia, where 7% of the onion (*Allium cepa*) plants tested were dually infected with these two distinct tospoviruses [24]. To investigate the nature and extent of interaction between two distinct tospoviruses in dually-infected plants, we utilized an experimental host system under controlled conditions wherein infection of a host plant with two distinct viruses and the subsequent intra-plant spread of infection was monitored. Datura (*Datura stramonium*) is a differential host for IYSV and TSWV. It is considered a permissive host for TSWV and a restrictive host for IYSV with respect to virus movement following infection (Figure 1). Following mechanical inoculation of datura, TSWV invades the plants and causes systemic infection by moving from the inoculated leaves to younger, uninoculated leaves. However, IYSV infection of datura remains localized to inoculated leaves [25]. Using virus species-specific probes for each of the viral genes, we show that in a mixed infection, TSWV facilitates the systemic movement of the silencing suppressor gene of a distinct tospovirus, IYSV, and results in more severe systemic symptoms compared to those produced by TSWV infection alone. This is the first ever experimental evidence of inter-species interaction at the genetic level facilitating systemic movement of a viral suppressor in an otherwise restrictive host. The observed phenomenon could be one of the mechanisms operating through which tospovirus diversity is generated and may explain the continued emergence of new tospoviruses and the expansion of the host range of this economically important group of plant viruses.

## Results

### Datura is a restrictive host for IYSV

**Figure 2:**
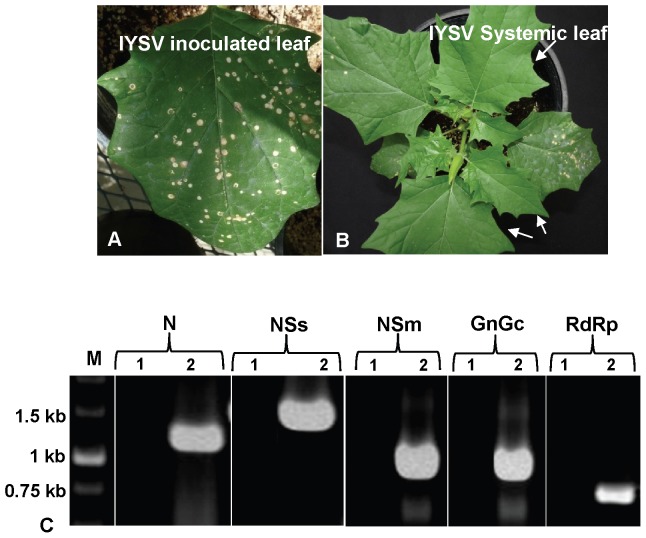
*Datura stramonium* is a restrictive host to infection by *Iris yellow spot virus* (IYSV). (A) Inoculated leaves with local lesions as a result of virus infection; (B) younger, uninoculated leaves of the same plant remain symptomless and virus-free; (C) Detection of IYSV genes in inoculated leaves of *D. stramonium* using reverse transcription-polymerase chain reaction. Total RNA from TSWV inoculated leaf (1) and healthy plant leaf as negative control (2) was used as a template for amplifying the genes using specific primers as described in Table 1. M: 1 kb Marker; N: IYSV nucleocapsid (N) protein gene; NSs: IYSV nonstructural (NSs) protein gene; NSm: IYSV nonstructural (NSm) protein gene; G_N_/G_C_: IYSV glycoprotein precursor (G_N_/G_C_); RdRp: IYSV RNA dependent RNA polymerase (RdRp).

When healthy *Datura stramonium* leaves are mechanically inoculated with IYSV, infection and subsequent symptom development remain confined to inoculated leaves. Leaves show 25 to 30 chlorotic local lesions of 2 to 5 mm 10 to 12 days post inoculation (DPI) (Figure 2A). By 20–25 DPI, the number of local lesions gradually increase and spread. As the lesions coalesce, the inoculated leaves dry up 35 to 40 DPI. Throughout this infection process, the virus remains localized and does not spread systematically to younger, un-inoculated leaves; and these younger and uninoculated ( = systemic) leaves remain symptomless and virus-free (Figure 2B). The virus could be detected only in the inoculated leaves by ELISA using the N protein-or NSs protein-specific antisera (Table 1). This was further confirmed by RT-PCR using N and NSs gene-specific primers that would produce 1.1 kb and 1.3 kb amplicons, respectively, which could be amplified from the total RNA from inoculated leaves, but not from the younger, un-inoculated leaves (Figure 2C, lanes 1 and 2). The presence of the remaining two RNAs of the IYSV genome, M and L RNAs, was confirmed by RT-PCR using primers specific to each of these two RNAs. The various coding regions (NSm, G_N_/G_C_ and RdRp) of these two RNAs could be amplified from inoculated leaves but not from uninoculated systemic leaves (data not shown); further confirming that IYSV was restricted to only inoculated leaves (Figure 2C, lanes 3, 4 and 5).

**Table 1:** Detection of *Iris yellow spot virus* (IYSV) and *Tomato spotted wilt virus* (TSWV) in *Datura stramonium* plants.

Treatment	Absorbance values A405*		
	IYSV		TSWV
	N	NSs	N
IYSV inoculated	1.96	0.79	N/A
IYSV systemic	0.12	0.20	N/A
TSWV inoculated	N/A	N/A	3.6
TSWV systemic	N/A	N/A	4.0
IYSV+TSWV inoculated	0.22	0.97	3.8
IYSV+TSWV systemic	0.38	0.65	4.0

* *D. stramonium* plants infected with IYSV or TSWV alone, or co-inoculated were tested for the presence of IYSV non structural protein (NSs) using direct antigen coated-enzyme linked immunosorbent assay, and the IYSV and TSWV nucleocapsid (N) proteins using double antibody sandwich enzyme-linked immunosorbent assay. N/A: not applicable as the antisera are specific to homologous antigens and do not cross react. The A405 values of uninfected (healthy) controls ranged from0.12 for 0.14 for all three antisera.

### Datura is a permissive host for TSWV

**Figure 3:**
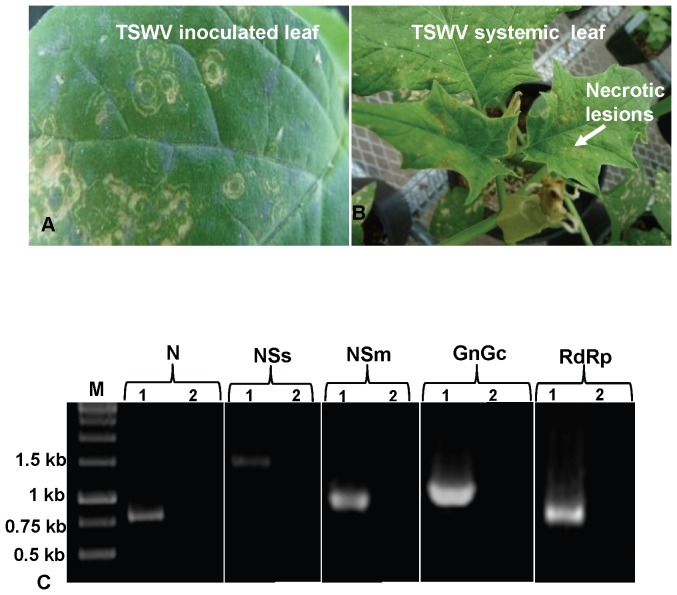
*Datura stramonium* is a permissive host for *Tomato spotted wilt virus* (TSWV). *D. stramonium* infected with TSWV (A) Inoculated leaves with local lesions as a result of virus infection; (B) Younger, uninoculated leaves of the same plant shows severe necrotic symptoms; (C) Detection of TSWV genes in inoculated leaves of *D. stramonium* using reverse transcription-polymerase chain reaction. Total RNA from TSWV inoculated leaf (1) and healthy plant leaf as negative control (2) was used as a template for amplifying the genes using specific primers as described in Table 1. M: 1 kb Marker; N: IYSV nucleocapsid (N) protein gene; NSs: IYSV nonstructural (NSs) protein gene; NSm: IYSV nonstructural (NSm) protein gene; G_N_/G_C_: IYSV glycoprotein precursor (G_N_/G_C_); RdRp: IYSV RNA dependent RNA polymerase (RdRp).

*D. stramonium* plants, when mechanically inoculated with TSWV, first show a few small concentric ring symptoms, 10–12 DPI on inoculated leaves (Figure 3A). These lesions increase in size and coalesce with others. Infection then spreads to younger and uninoculated leaves ( = systemic leaves). Systemic symptoms include necrotic spots, curling, yellowing, greening and mottling that spread rapidly throughout the plant (Figure 3B). The emerging buds, flowers and fruits also show severe necrosis (Figure 3B), ultimately leading to senescence of the infected plants by 60–80 DPI. Virus could be detected by ELISA 10–12 DPI in both inoculated and younger, uninoculated leaves. To further confirm that the virus became systemic in datura, the presence of all three genomic RNAs was tested in both inoculated and uninoculated symptomatic leaves. Genes encoded by S (N and NSs), M (NSm and G_N_/G_C_) and L (RdRp) RNA were amplified using gene-specific primers. Both inoculated (Figure 3C) and systemic leaves were found to contain all five genes. The resulting amplicons were cloned and sequenced to confirm the systemic nature of TSWV infection in datura.

### Datura becomes permissive host for IYSV in the presence of TSWV

**Figure 4:**
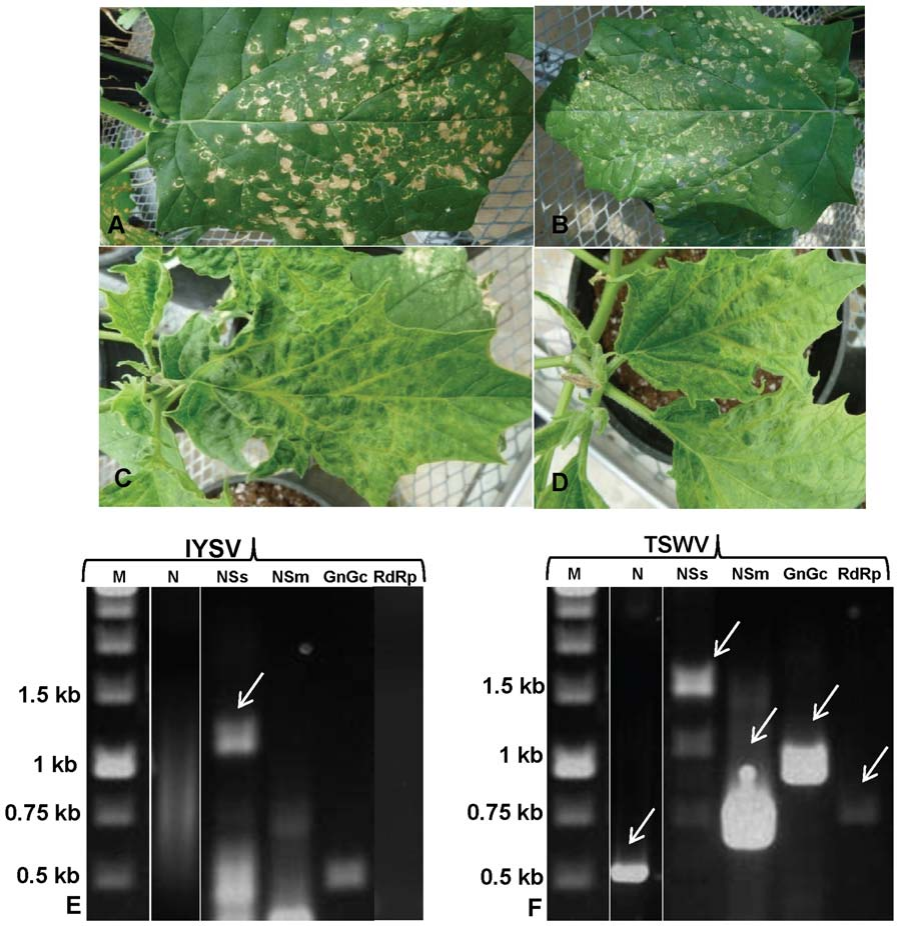
*Datura stramonium* plants co-inoculated with *Iris yellow spot virus* (IYSV) and *Tomato spotted wilt virus* (TSWV). (A) Leaves inoculated with both IYSV and TSWV Dual infection results in more severe systemic symptoms, necrotic spots and concentric rings on inoculated leaves; (B) systemic leaves from dual inoculated plants showing symptoms including leaf curling, severe venial chlorosis, and yellowing on younger un-inoculated leaves; (C) Detection of IYSV genes in systemic leaves of *D. stramonium* co-inoculated with TSWV and IYSV using reverse transcription-polymerase chain reaction., (D) Detection of TSWV genes in systemic leaves of *D. stramonium* co-inoculated with TSWV and IYSV using reverse transcription-polymerase chain reaction; Lane M: Marker; N: nucleocapsid (N) protein gene; NSs: nonstructural (NSs) protein gene; NSm: nonstructural (NSm) protein gene; G_N_/G_C_: glycoprotein precursor (G_N_/G_C_); RdRp: RNA dependent RNA polymerase (RdRp);.

When leaves of healthy datura were mechanically inoculated with a mixture of IYSV and TSWV, symptoms first appeared as small chlorotic spots, which gradually developed into mixtures of necrotic spots and concentric rings 7–10 DPI (Figure 4A, B). Symptoms subsequently spread throughout the inoculated leaves and then spread to younger, uninoculated leaves resulting in systemic infection. Systemic symptoms appeared 15–20 DPI that included severe curling, mottling, yellowing and greening (Figure 4C, D). The newly emerging leaves as well as buds showed severe necrotic symptoms and plant growth was severely affected. The symptoms in co-infected plants were much more severe (Figure 4) as compared to plants inoculated with TSWV or IYSV only (Figure 2 and 3). The plants also succumbed faster to the dual infection and senesced by 45–50 DPI, as compared to those with TSWV alone which survived 80 DPI.

### Detection of IYSV and TSWV in inoculated leaves of dually inoculated datura

The inoculated and systemic leaves of dually inoculated datura plants were separately tested for the presence of both viruses 10–12 DPI by ELISA and RT-PCR. Both IYSV N and TSWV N proteins could be detected in the inoculated leaves by ELISA. As TSWV becomes systemic in datura, TSWV N could also be detected in the systemic leaf. As IYSV infection remains localized to the inoculated leaf in *D. stramonium*, IYSV N could not be detected in the systemic leaves. However, interestingly, the IYSV NSs protein was detected by ELISA in the systemic leaves of dually inoculated plants using NSs-specific antisera (Table 1).

The uninoculated younger, systemic leaves of dually inoculated datura plants with severe curling and mottling were tested for the presence of S, M and L RNAs of IYSV and TSWV by RT-PCR using gene-specific primers (Table 2). As expected, the systemic leaves of a dually inoculated plant tested at 25–30 DPI showed the presence of all five genes of TSWV (Figure 3F). In the case of IYSV in the same samples, N, NSm, G_N_/G_C_ or RdRp genes could not be detected. Interestingly, of all the IYSV-coded genes, only the NSs gene was amplified (Figure 3E). All the genes that could be amplified were cloned and sequenced to confirm the identity of each amplicon.

**Table 2:** List of primers used in reverse transcription-polymerase chain reaction.

RNA	Primer pairs	IYSV	Gene	Size bp	Tm
S RNA	IYSV-N-F	CTCTTAAACACATTTAACAAGCA	N	1100	55
	IYSV-N-R	TAAAACAAACATTCAAACAA			
	IYSV-NSs-F	CCTTTTTTTTTTCATATGTCTACCGTTAGGACTACGGC	NSs	1330	65
	IYSV-NSs-R	TTATGGATCCTCACTGCAGCTCTTCTACA			
M RNA	IYSV-NSm1F	ATGTCTCTCCTAACTAACGTG	NSm	994	55
	IYSV-NSm994C	CATACTTCATTAAATCTGTTCT			
	IYSV-G_N_/G_C_-3790F	CATTTTTTGTCTTCCAAAGT	G_N_/G_C_	1016	55
	IYSV-G_N_/G_C_-4806C	CAAACAATCAGCCTAAGATG			
L RNA	IYSV-RdRp-4870F	ATACAATGTCAAGCACTTAG	RdRp	711	52
	IYSV-RdRp-5581C	TGTACAGATTGGTTAGTATG			
		**TSWV**			
S RNA	TSWV-N-1F	ATGTCTAAGGTTAAGCTCACTA	N	777	64
	TSWV-N-777C	TTAAGCAAGTTCTGTGAGTTTT			
	TSWV-N-248F	CAGACAGGATTGGAGCCACT	N	462	60
	TSWV-N-710C	TCACTGTAATGTTCCATAGCAA			
	TSWV-NSs-1F	AGAGCAATTGTGTCATAATT	NSs	1512	55
	TSWV-NSs-1531C	TTATAAGTAAAGAAAGAAAA			
M RNA	TSWV-NSm-1F	ATGTTGACTCTTTTCGGT	NSm	909	55
	TSWV-NSm-909C	CTATATTTCATCAAAGGATAA			
	TSWV-G_N_/G_C_-3050F	CCTGTATAATCCGAAAACCC	G_N_/G_C_	976	52.5
	TSWV-G_N_/G_C_-4025C	GCATCACTAGCCCTGAG			
L RNA	TSWV-RdRp-4893F	TCCTGGTGAAGTGAATGATA	RdRp	780	52
	TSWV-RdRp-5883C	AAACCACCTGAAATTGTAGT			

### Expression of N and NSs genes in dually infected datura plants

**Figure 5:**
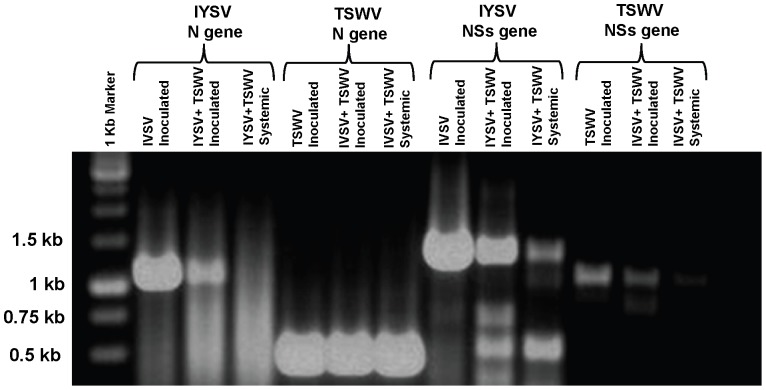
Detection of nucleocapsid (N) gene and non-structural (NSs) genes in inoculated and uninoculated systemic leaves of *Datura stramonium* plants infected with *Iris yellow spot virus* (IYSV) and *Tomato spotted wilt virus* (TSWV) in plants inoculated with either IYSV, TSWV or both.

**Figure 6:**
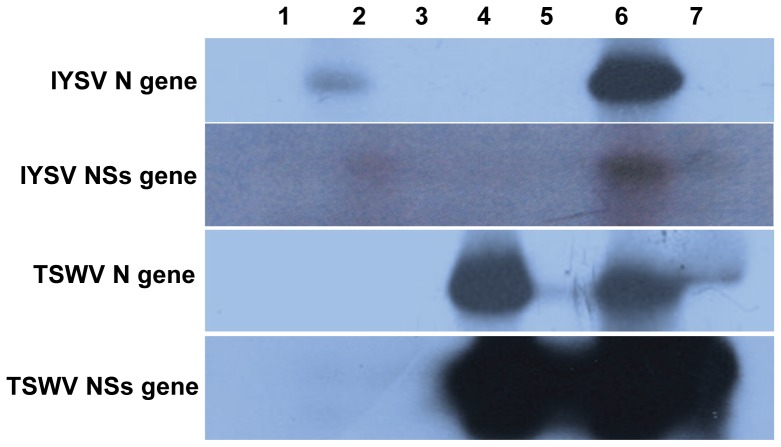
Expression of N and NSs genes of *Iris yellow spot virus* (IYSV) and *Tomato spotted wilt virus* (TSWV) in single or dually infected *Datura stramonium* plants using gene-specific cDNA probes. Leaf samples were harvested 15–20 days post inoculation from inoculated and systemic leaves. Lanes: 1-Healthy, uninfected control; 2-IYSV only inoculated leaf; 3-IYSV only systemic leaf; 4-TSWV only inoculated leaf; 5-TSWV only systemic leaf; 6-IYSV+ TSWV-inoculated leaf; 7-IYSV+ TSWV systemic leaf.

RT-PCR with specific primers showed that TSWV N and NSs could be detected in inoculated leaves of single and co-infected plants, as well as systemic leaves of a co-infected plant (Figure 5). IYSV N and NSs were also expressed in inoculated leaves of single- or dually- inoculated plants; however, only IYSV NSs was expressed in systemic leaves of a co-infected plant (Figure 5). All the genes that could be amplified were cloned and sequenced to confirm the identity of each amplicon. To determine the relative levels of the viral genomic RNA transcripts, the inoculated and systemic leaves of *D. stramonium* from single and dually infected plants (IYSV+TSWV) were analyzed for IYSV and TSWV N and NSs gene expression using gene-specific cDNA probes (Figure 6). The results confirm the specificity of the probes for IYSV and TSWV as no bands were detected for IYSV when probed with TSWV and vice versa. In agreement with the results obtained with RT-PCR, the IYSV N gene expression could be detected in inoculated leaves of IYSV and was absent in systemic leaves (Figure 6A) in plants inoculated with IYSV only. Again, in agreement with the RT PCR results, in the case of plants inoculated with both IYSV and TSWV, the IYSV N gene was detected only in inoculated leaves (Figure 6A), and was absent in the younger, uninoculated leaf. Moreover, Northern blots showed that the IYSV N gene expression was higher in inoculated leaves in case of dually infected plants as compared to IYSV alone (Figure 6A).

In the case of IYSV NSs, the gene expression was detected only in inoculated leaves of singly infected plants and was absent in systemic leaves (Figure 6B), which was in agreement with the results obtained by RT-PCR. However, in the case of dually infected plants, NSs transcript was detected in both inoculated and systemic leaves (Figure 6B), indicating that only IYSV NSs is selectively expressed in systemic leaves in the presence of TSWV.

As expected, the TSWV N and NSs gene expression was detected in inoculated and systemic leaves of plants with single and dual infection (Figure 6C, D), and the N gene expression pattern of TSWV was similar in both single and dually infected plants, with expression being higher in inoculated leaves as compared to systemic leaves (Figure 6C). However, TSWV NSs expression showed variation in the presence of IYSV. The relative level of TSWV NSs was much higher in systemic leaves of dually infected plants (Figure 6D) compared to leaves from plants infected with TSWV only. These results showed that the IYSV NSs gene is expressed only in systemic leaves of plants inoculated with both IYSV and TSWV, and also that the expression of TSWV NSs in systemic leaves is much higher in the presence of IYSV NSs.

### Small RNA expression of N and NSs genes

**Figure 7:**
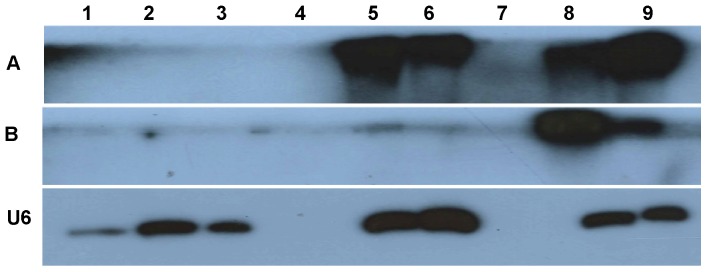
Small interfering (si) RNAs of N and NSs genes of *Tomato spotted wilt virus* (TSWV) in single or dually infected *Datura stramonium* plants. The leaf samples were harvested 25–30 days after inoculation from inoculated and systemic leaves A: TSWV N gene specific small interfering (Si) RNAs B: TSWV NSs gene-specific siRNAs, U6: Ubiquitin as loading control for RNA. Lanes: 1-Healthy, uninfected control; 2- IYSV-inoculated leaf; 3- IYSV systemic leaf; 4-Blank; 5-IYSV+ TSWV-inoculated leaf; 6-IYSV+ TSWV systemic leaf; 7-Blank; 8-TSWV-inoculated leaf; 9-TSWV systemic leaf.

Small RNAs for N and NSs genes were analyzed by Northern blot analysis for single and dual infections in inoculated as well as systemic leaves. The small RNA fraction from infected samples was subjected to polyacrylamide gel electrophoresis and Northern hybridization using gene-specific probes to detect the presence of 21–24 nucleotide siRNAs. TSWV N gene-specific siRNAs were detected in both inoculated and systemic leaves of plants in the case of single as well as dual infection (Figure 7A). The probes used were specific for TSWV and did not hybridize with any of the samples from IYSV-inoculated plants (Figure 7A, lanes 2, 3). In the case of NSs, interestingly, we could detect the siRNAs in leaf samples inoculated with TSWV only (Figure 7B, lanes 8, 9); while we could not detect TSWV NSs siRNAs either in the inoculated or systemic leaves of plants inoculated with both TSWV and IYSV (Figure 7B, lanes 5, 6). This suggested that the silencing suppressor (NSs) of TSWV was not subjected to the plant's silencing machinery in the presence of silencing suppressor of IYSV. The blots were also probed with IYSV N- and NSs-specific probes and we could not detect siRNAs for either gene in single or dually inoculated plants (data not shown).

## Discussion

Mixed infections of plants with viruses is a natural phenomenon, and the outcome of this inter-virus interaction is influenced by several factors including the host plant's genetic background and growth stage, genetic relatedness of the viruses, and environmental conditions. The outcomes include elimination of one virus from the mixed infection by the second virus, co-existence and continued replication of both viruses for the remainder of the plant's life, or increased titer of one virus resulting in more severe symptoms (synergism). Mixed infections could also result in genetic recombination or genetic reassortment between the co-infecting viruses that could lead to generation of new viruses or strains with a subsequent expansion of the host range [26]–[28]. Recently Webster et al., [15] reported emergence of a new tospovirus *Groundnut ring spot virus* (GRSV) in the USA due to genome reassortment of two viruses, GRSV and *Tomato chlorotic spot virus* (TCSV), with the large (L) and small (S) RNAs coming from GRSV and the medium (M) RNA from TCSV (i.e., L_G_M_T_S_G_). This exchange of genetic material and interaction between co-infecting viruses resulting in synergism or complementation could lead to changes in biological properties and increased diversity of viruses, especially those with segmented genomes due to genetic reassortment [21]. A related phenomenon is transcomplementation, in which a viral protein, usually expressed from a transgene, enhances or supports the infection of another virus from a distinct species [29].

Synergistic interactions between viruses leading to more severe disease have been described for positive-stranded RNA viruses and DNA viruses [30]–[39]. Although synergism and transcomplementation are very common among viruses, there are no reports of ambisense or negative sense viruses showing this phenomenon [29]. We present the first report of genetic complementation in ambisense tospoviruses. Our finding that TSWV is potentially facilitating the movement of a viral protein of another distinct tospovirus, IYSV, from point of inoculation to younger, systemic leaves of an otherwise restrictive host is the first instance of such an interaction between viruses in the genus *Tospovirus*.

We utilized a model system wherein the experimental host, datura, responds differentially to two distinct tospoviruses, IYSV and TSWV. While this host is susceptible to infection by both viruses, it is a permissive host to TSWV (where the virus invades the plant and causes systemic infection), whereas IYSV infection is confined to inoculated leaves and fails to move to younger, uninoculated leaves [25]. There are more than 24 distinct tospoviruses known to date and mixed infections of tospoviruses are taking place under natural conditions [23]–[24]. However, there has been so far no experimental evidence of genetic complementation between two distinct tospoviruses. The differential response of datura to IYSV and TSWV allowed us to investigate if such an interaction at the genetic level takes place in dually infected plants.

Datura plants inoculated with IYSV alone showed symptoms of infection only on the inoculated leaves, with younger systemic leaves remaining symptomless. On the other hand, TWSV inoculation resulted in symptoms on the inoculated as well as systemic leaves (Figure 3). This was also corroborated in terms of gene expression, with young systemic leaves in the case of plants inoculated with IYSV remaining negative for all genes tested and TSWV showing positive gene expression of all three RNAs (S, M and LRNA) in inoculated as well as systemic leaves.

Dual infection of datura plants with IYSV and TSWV resulted in an increase in the symptom severity of the inoculated as well as the systemic leaves with plants succumbing to the virus 40 days post-inoculation (dpi) as compared to 80 dpi in the case of single infection with TSWV. In addition, the IYSV NSs was selectively expressed in the younger, uninoculated leaves of dually infected *D. stramonium*. NSs have been reported as the silencing suppressor in tospoviruses [20].

Availability of antisera specific to each of the TSWV and IYSV N proteins and the IYSV NSs protein enabled us to determine the expression of these genes in individually and dually infected plants. While the relative levels of these proteins as reflected by the absorbance values varied among individual plants within a treatment, we were able to consistently detect these proteins. TSWV N could be detected in inoculated as well as systemic leaves as expected. IYSV N and NSs could be found only in inoculated leaves in singly infected plants (since the virus is confined to inoculated leaves in datura). In case of dually infected plants, the IYSV NSs protein was expressed in uninoculated leaves, albeit to a lower level.

Hassani- Mehraban [40] obtained transgenic plants resistant to five different tomato-infecting *Tospovirus* spp. including TSWV and a tomato-infecting strain of *Tomato yellow ring virus* (TYRV-t) using partial N gene sequences. They showed that transgenic resistance against TYRV-t does not hold against the soybean strain (TYRV-s) and is broken by TYRV-t when co-inoculated with TYRV-s. TYRV-t could be detected in the systemic leaves of a resistant line in co-inoculated plants implying a transcomplementation event involving a protein of TYRV-s. They further showed that the presence of TYRV-s encoded silencing suppressor (NSs) was crucial for rescuing of TYRV-t. The severe symptoms in datura plants dually infected with TSWV and IYSV, compared to those individually infected either with TSWV or IYSV, could be explained by the fact that the level of the silencing suppressor, NSs, of both viruses was much higher in dually infected than in singly infected Datura (Figure 7). Even in the case of TSWV, which causes systemic infection in datura, the presence of IYSV in the same plant resulted in more severe systemic symptoms. This could be due to the selected movement and subsequent accumulation of NSs of IYSV in the younger, uninoculated leaves. This is the first experimental evidence of synergistic interactions between two distinct viruses in the genus *Tospovirus.*

TSWV NSs has been shown to be a strong suppressor of RNA silencing not only in plants but also in Tick cells [41]. Oliveira et al., [42] reported that NSs of TSWV enhanced baculovirus replication in permissive and semi-permissive insect cell lines. A recombinant baculovirus with TSWV NSs was inserted into the viral genome and the construct replicated to higher titers than the wild type in permissive and semi-permissive lepidopteron insect cell lines tested. In addition, it also infected a non-permissive host cell line in the presence of NSs. This suggests that NSs plays an important role in enhancement of gene expression during tospovirus infection in its thrips vector. In our study, we found that TSWV NSs facilitated the selective expression of IYSV NSs in the systemic leaves of a dually infected plant. Also, the presence of the NSs from both viruses in inoculated as well as systemic leaves enhanced the severity of symptoms as well as virus titers. The N as well as NSs gene expression of IYSV in inoculated leaves of datura was enhanced in the presence of TSWV (Figure 6). The TSWV NSs expression was also higher in the systemic leaf of a dually infected plant. These results are of significance in terms of field infections by multiple tospoviruses even in restricted or non-permissive hosts. It was recently shown that NSs encoded by GBNV is a bifunctional enzyme and could participate in viral movement, replication and suppression of host defense mechanism [43]. One experimental approach to verify the observed interaction between the silencing suppressors is to use NSs-deficient mutant TSWV or IYSV. However, lack of infectious clones of these viruses is a constraint to carrying out such an experiment.

In terms of small RNA expression in single versus dually infected datura plants, it was observed that systemic leaves of dually infected plants showed reduced levels of TSWV N gene specific small interfering RNA (siRNAs) (Figure 7, compare lanes 6 and 9). No TSWV NSs-specific siRNAs were detected in inoculated nor systemic leaves of dually infected datura plant (Figure 7, lanes 5 and 6 compared to lanes 8 and 9) indicating a more efficient suppression of host silencing machinery in the presence of NSs from both viruses as compared to the presence of only TSWV NSs. Also, we could not detect siRNAs of IYSV N or NSs genes in single as well as dually infected plants. This suggests that the IYSV NSs is a strong suppressor of RNA silencing and could be functioning by sequestering the siRNAs or may also have affinity for long dsRNAs. Schnettler et al., [44] showed that NSs from different tospoviruses interfere with RNA silencing pathway by sequestering siRNAs and microRNA duplexes. In addition, NSs from TSWV, INSV and GRSV was shown to have affinity for long dsRNA, whereas TYRV NSs did not. It was suggested that TSWV NSs may bind to the ambisense S-RNA encoded hairpin structure to prevent its recognition and subsequent degradation in plants by Dicer-like proteins, while simultaneously supporting translational enhancement of viral transcripts by circularization [45]. Our results also show that NSs of IYSV and TSWV in a dually infected plant may have escaped the plant RNA silencing machinery as indicated by absence of NSs-specific siRNAs in inoculated as well as systemic leaves.

Genetic reassortment has long been accepted as one of the means by which new viruses or strains are generated among viruses with segmented genomes. Such a naturally occurring genetic reassortant between GRSV and *Tomato chlorotic spot virus* (TCSV) has recently been described [15]. Tobacco plants infected with *Tobacco mosaic virus* and *Cucumber mosaic virus* produce a synergistic disease with severe malformations [46]. They have further shown that the effect appears to be a result of joint action of the viral silencing suppressors, CMV 2b protein and TMV162kDa replicase subunit, on the plant silencing machinery. Our findings suggest that the genetic complementation could result in expanding the host range of a particular virus by turning a restrictive host into a permissive one. Hassani Mehraban et al., [40] showed that isolates with even less than 20% sequence divergence can break down RNA silencing-mediated resistance to tospoviruses as a result of NSs expression. This has consequences for the use of RNA-mediated resistance against *Tospovirus* spp. such as TYRV and IYSV with N gene variation of only 10 to 15%.

The model system we developed facilitates further studies on the nature of protein-protein interactions between the silencing suppressors of distinct tospoviruses in dually infected plants, the nature of the plant factors that interact with the viral proteins, and the effect of dual infection on the profile of virus-specific small RNAs produced as a result of host-induced gene silencing.

## Materials and Methods

### Viruses

IYSV and TSWV, originally isolated from naturally infected onion and peanut (*Arachis hypogaea*) plants, were maintained on experimental hosts, *Datura stramonium* and *Nicotiana benthamiana* under controlled conditions in a greenhouse.

### Virus inoculations of host plants

Virus-infected tissue was homogenized by grinding in 0.01 M sodium phosphate buffer (pH 7.0) containing 0.4% β-Mercaptoethanol and the homogenate was used as the inoculum for mechanical inoculation to experimental hosts. Fully expanded leaves (25–30 days old) of *D. stramonium* and *N. benthamiana* were dusted with silicon carbide (600 mesh) and the homogenate was manually applied using cotton buds. Inoculated plants were maintained at 25/18°C (day/night), 14 h day light and observed for development of symptoms. For establishing mixed infections, leaves from *D. stramonium* plants individually infected with TSWV or IYSV were homogenized together in equal amount of tissue and mechanically inoculated on to healthy *D.stramonium* and *N. benthamiana* plants as stated earlier. All experiments were repeated at least three times. The inoculated as well as systemic leaves of both hosts were harvested 25–30 days after inoculation, and stored at 4°C for further analysis.

### Enzyme-linked immunosorbent assay (ELISA)

Virus infection of inoculated plants was confirmed by ELISA using commercially available kits for TSWV and IYSV (Agdia Inc., Elkhart IN USA) following manufacturer's instructions. IYSV NSs-specific polyclonal antiserum was made to *E. coli*-expressed NSs protein which was used in a direct antigen-coated ELISA format [47].

### Reverse transcription polymerase chain reaction (RT-PCR)

Total plant RNA was extracted separately from the symptomatic and asymptomatic IYSV and TSWV-infected plant tissue using the Plant RNeasy mini kit (Qiagen, Valencia CA USA). RT-PCR was performed to detect the presence of various genomic RNAs (L, M, and S) of TSWV and IYSV using gene-specific primers (Table 2).

### RNA extraction and analysis

Symptomatic tissues were collected from inoculated and systemic leaves from co-infected datura plants and snap frozen immediately in liquid nitrogen. Frozen leaf tissue was homogenized in liquid nitrogen using a mortar and pestle. Total RNA was extracted using TRIZOL reagent (Life Technologies, Green Island NY USA) following the manufacturer's instructions. Low molecular weight RNAs were selectively precipitated by PEG_8000_/NaCl as described previously [48], except that the low molecular weight RNA was resuspended in 100% deionised formamide. The concentration of low molecular weight RNA was determined using Nano Drop spectrophotometer (Thermo Fisher Scientific, Barrington IL USA) and visualized on agarose gel to enable equal loading.

Total RNA (20 ug) from plant samples was subjected to Northern blot analysis [49]. RNA blots were probed with full-length TSWV N and NSs and IYSV N and NSs genes radiolabeled with ^32^P dATP. Small RNA was separated on a 17% polyacrylamide- 7M urea gel and transferred to positively charged nylon membrane (Roche, San Francisco, CA USA) by the semi-dry blot method [50]. Hybridization was done at 40°C using UltraHyb buffer (Life Technologies, Green Island, NY USA) and ^32^P-radiolabeled probes by end-labelling with T4 polynucleotide kinase enzyme for oligo probes and random-priming method with Megaprime kit (GE Healthcare, USA) for larger double-stranded DNA probes following manufacturer's instructions. The radiolabeled probes were column-purified. The blots were washed twice in 2× SSC, 0.1% SDS at 40°C for 10 minutes, exposed to X-ray film (Agfa-Curix Ortho HT-G, Mortsel, Belgium), and the film was developed. Blots were stripped in boiling 2× SSC, 0.1% SDS for 10–15 minutes, and verified for residual radiation by overnight exposure before re-probing with a different probe.
